# Comparison of workers’ compensation systems in Singapore and Japan

**DOI:** 10.1093/joccuh/uiag038

**Published:** 2026-07-12

**Authors:** Kee Leng Chua, Alvin Kian Wei Tan, Kimiyo Mori, Chikage Nagano, Seichi Horie, David Soo Quee Koh

**Affiliations:** MOH Holdings Pte Ltd, 1 North Buona Vista Link, #09-01 Elementum, Singapore 139691, Singapore; Saw Swee Hock School of Public Health, National University of Singapore, Singapore; Saw Swee Hock School of Public Health, National University of Singapore, Singapore; Department of Occupational and Environmental Medicine, Singapore General Hospital, Singapore; Department of Health Policy and Management, Institute of Industrial Ecological Sciences, University of Occupational and Environmental Health, Fukuoka, Japan; Department of Health Policy and Management, Institute of Industrial Ecological Sciences, University of Occupational and Environmental Health, Fukuoka, Japan; Department of Health Policy and Management, Institute of Industrial Ecological Sciences, University of Occupational and Environmental Health, Fukuoka, Japan; Saw Swee Hock School of Public Health, National University of Singapore, Singapore

**Keywords:** workers’ compensation, legislation, Singapore, Japan, occupational health

## Abstract

**Objectives:**

Singapore and Japan have well-established workers’ compensation systems that differ significantly in their structure and approach. We sought to identify key differences between the 2 systems, highlight successful practices for consideration, and critically assess the advantages and disadvantages of each approach.

**Methods:**

A comparative analysis of workers’ compensation systems in Singapore and Japan was conducted in the form of a narrative review, drawing upon published data from peer-reviewed scientific journals, official national sources, and key international organizations.

**Results:**

Singapore’s workers’ compensation system focuses on ensuring employer competitiveness while providing workers with basic financial protection and limiting spillover costs to society through tightly scoped compensation coverage. In contrast, Japan’s workers’ compensation system prioritizes worker health with more comprehensive compensation benefits, coverage, and clearer insurance mechanisms to recognize the true costs of poor workplace safety and health. Both Singapore and Japan face similar challenges for workers’ compensation in terms of apportioning attributability of occupational diseases and underreporting of work-related incidents.

**Conclusions:**

A better understanding of the underlying principles and trade-offs within each workers’ compensation system allows for identification of opportunities to strengthen fairness and ensure more equitable outcomes for workers, employers, and society. Both Singapore and Japan can benefit from closer collaboration to share best practices, innovative solutions, and research efforts to address shared challenges.

## Teaser

**Key points**
 

- **What is already known on this topic:** Singapore and Japan are high-income economies in the Asia-Pacific region with well-established workers’ compensation systems adopting a principle of no-fault liability. Workers’ compensation systems allow workers to claim for work-related injuries or diseases without the need for torts and have been viewed as a quicker and lower cost alternative.
- **What this study adds:** Comparative analyses of workers’ compensation systems in Asian high-income economies such as Singapore and Japan remain limited. This study compares both countries’ coverage and eligibility for workers’ compensation, benefits covered under workers’ compensation, claims process, compensation statistics, and the role of the occupational health physician in workers’ compensation.
- **How this study might affect research, practice, or policy:** This study presents an opportunity to better understand the potential consequences of different policy choices and provide valuable insights in the ways they affect outcomes for workers, employers, and society. The study discusses and provides further insight into how policy design affects the distribution of costs and responsibilities between workers, employers, and society. Understanding the underlying principles and trade-offs in each system allows for identification of opportunities to strengthen fairness and ensure more equitable outcomes.

## 1. Introduction

Singapore and Japan are high-income economies in the Asia-Pacific region with well-established workers’ compensation systems that adopt a principle of no-fault liability. Both countries face similar challenges, including an aging workforce, evolving employment arrangements such as platform work, and persistent underreporting of work-related illnesses. Despite the shared foundations and challenges, the 2 systems differ in their underlying social welfare philosophies and how workers’ compensation systems are structured.

### 1.1 History and evolution of compensation systems in Singapore and Japan

#### 1.1.1 Singapore

Workers’ compensation legislation was first passed in the Straits Settlements in 1932 as the Workmen’s Compensation Ordinance under British colonial administration, stipulating no-fault compensation to workmen for injuries or death arising out of or in the course of employment.^1^ After independence in 1965, rapid industrialization contributed to an increase in workplace accidents and fatalities, leading to the Workmen’s Compensation (Amendment) Act passed in 1971, which built on the Workmen’s Compensation Ordinance to provide compensation to a broader group of lower-wage workers beyond workmen.^2^

In 2004, three major workplace accidents occurred in Singapore: the Nicoll Highway collapse, a fire onboard the vessel “Almudaina,” and an accident at the Fusionopolis worksite.^3–5^ This series of high-profile workplace accidents contributed to the reform of Singapore’s workplace safety and health (WSH) landscape, culminating in the enactment of the Workplace Safety and Health Act in 2006, which covered all workplaces. Correspondingly, the Workmen’s Compensation Act was updated to the Work Injury Compensation Act (WICA), expanding compensation to almost all employees, except for uniformed personnel and domestic workers.^6^ From January 1, 2025, the Platform Workers Act was introduced and extended coverage of workers’ compensation to all platform workers.

#### 1.1.2 Japan

The origins of workers’ compensation for occupational accidents in Japan date back to 1872, when government enterprises provided lump-sum payments for work-related deaths or injuries, and medical benefits on a charitable basis, reflecting the traditional concept of relief for the economically distressed (*jutsu*). This approach evolved into formal assistance obligations (*fujo*) and extended to occupational diseases under the Mining Ordinance of 1890 and the Factory Act of 1911.^7^^,^^8^

In 1947, the Labour Standards Act (LSA) established compensation for work-related injuries and illnesses as a statutory right (*hoshō*) based on employers’ no-fault liability, while the Industrial Accident Compensation Insurance Act (IACIA) ensured prompt and equitable benefits to mitigate employers’ burdens. From its inception in 1947, the system applied broadly across industries, with exclusions primarily based on employment status rather than sector, such as domestic workers and parts of the self-employed and small-scale sectors. Additional reforms expanded its functions, including experience-rated premiums (1951), benefits for long-term medical treatment (1960), special enrollment schemes (1965),^9^ coverage of commuting accidents (1973), integration of the Seamen’s Insurance scheme (2010), and progressive extension of special enrollment to freelancers from 2020 onwards.^10^

**Figure 1:**
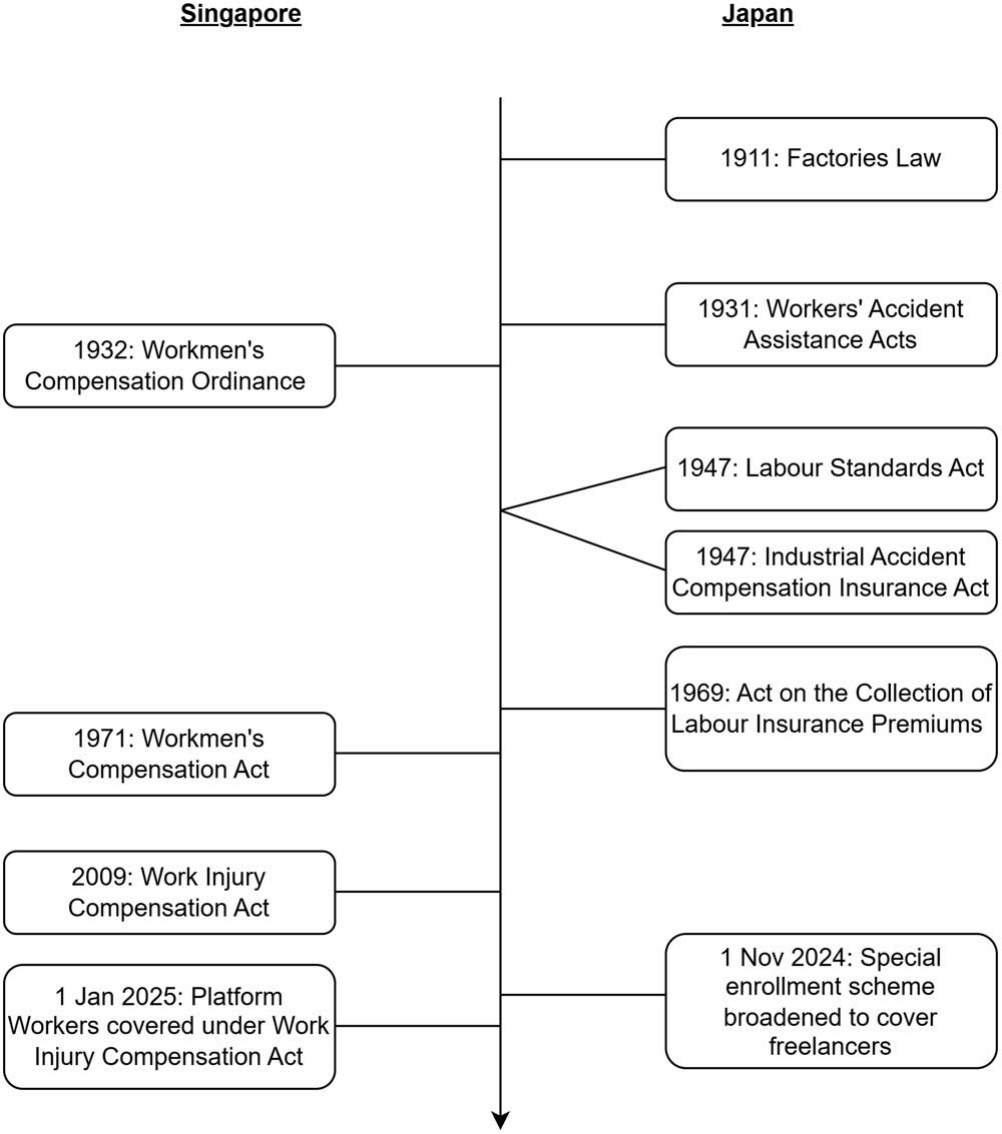
Key changes and evolution of workers’ compensation laws in Singapore and Japan.

Figure 1 summarizes the main events and evolution of Singapore’s and Japan’s compensation systems.

#### 1.1.3 Aims

This review aimed to identify key differences between the workers’ compensation systems in Singapore and Japan, highlight successful practices, and critically assess the advantages and disadvantages of each approach to identify opportunities to strengthen fairness and promote more equitable outcomes for workers, employers, and society.

## 2. Methodology

A comparative analysis of workers’ compensation systems in Singapore and Japan was conducted in the form of a narrative review. The review drew upon peer-reviewed literature identified on PubMed and Google Scholar using search terms including “compensation,” “workers’ compensation,” “industrial accident compensation,” “workers’ compensation insurance.” “industrial accident compensation insurance,” “Singapore,” and “Japan”. Official publications from governmental agencies, national organizations, and key international organizations were reviewed, including reports from Singapore’s Ministry of Manpower (MOM), Japan’s Ministry of Health, Labour and Welfare (MHLW) and the International Labour Organization. The narrative review methodology was informed by the Scale for the Assessment of Narrative Review Articles.^11^

A comparison of both countries’ coverage and eligibility for workers’ compensation, benefits covered under workers’ compensation, claims process, compensation statistics, and the role of the occupational health physician in workers’ compensation was conducted. For the analysis of compensation statistics, the number of workers’ compensation claims awarded, or new beneficiaries, was normalized per 100 000 covered workers. For Japan, covered workers comprised workers insured under the Industrial Accident Compensation Insurance. For Singapore, the number of employed persons excluding self-employed persons and migrant domestic workers was used as a proxy for workers covered under the WICA, as this information was not publicly available. Compensation amounts were converted to US dollars (USD) using the official annual period-average exchange rate for the respective year.

## 3. Results

### 3.1 Current state of workers’ compensation

#### 3.1.1 Singapore

The WICA is Singapore’s primary legislation governing workers’ compensation.^12^ The purpose of the WICA is to provide payment for employees and platform workers for injuries or occupational diseases arising out of and in the course of employment, and also to regulate providers of insurance for liability under the Act.^13^ Importantly, workers who are injured or fall ill at work can either claim under the WICA or common law, but not both.^14^

Key subsidiary legislation under the WICA includes the Work Injury Compensation Regulations, Work Injury Compensation (Insurance) Regulations, and Work Injury Compensation (Workers’ Fund) Regulations.^15–17^ Legislation pertaining to other aspects of work such as wages, working hours, leave entitlements, and protection from wrongful dismissal is covered under a separate piece of legislation, the Employment Act.

#### 3.1.2 Japan

The LSA, IACIA, and Act on the Collection, etc. of Insurance Premiums of Labor Insurance (ACIPLI) are Japan’s main legislation governing workers’ compensation. Under the LSA, the employer is required to compensate workers for medical treatment, wages for temporary absence from work, disabilities, and other requirements for work injuries and illnesses. In addition to compensation for injury or illness in the course of employment, the LSA regulates other aspects of work such as labor contracts, wages, and working hours.

The IACIA provides financial protection, through an industrial accident compensation insurance scheme operated by the state, to workers who are injured, become ill, or are disabled in the course of their duties or commute for work.^18^^,^^19^ Compulsory work injury compensation insurance is mandated through the ACIPLI, which outlines the establishment of labor insurance relationships, calculation of premiums, and procedures for premium collection.

When accident compensation is provided under the IACIA, employers are exempt from providing compensation under the LSA.^20^ Workers or surviving family members can claim compensation for the same incident under both the IACIA and common law. However, the total amount of damages and compensation is adjusted, and employers are exempt from liability under the Civil Code up to the compensation amount provided by the IACIA.^19^

Table 1 summarizes the statutory requirements in relation to workers’ compensation for both Singapore and Japan.

**Table 1:** Summary of workers’ compensation laws in Singapore and Japan.

Country	Legislation	Statutory requirements
**Singapore**	Work Injury Compensation Act	Liability for compensation: Employers and platform operators must compensate employees or platform workers for work injuries or occupational diseases arising from the course of employment Provision of workers’ compensation insurance: Employers and platform operators must maintain insurance policies that are approved under the Work Injury Compensation Act 2019 Claims and compensation procedures: Methodology for the computation of compensation benefits stated
	Work Injury Compensation Regulations	Claims and compensation procedures: Employers and platform operators must notify the Commissioner and their respective insurers within specified timeframes (10 days) after becoming aware of an accident or occupational disease Employers, insurers, and platform operators must make payments for compensation within a specified timeframe (generally 14 to 21 days) after receiving necessary documents or directives
	Work Injury Compensation (Insurance) Regulations	Persons requiring workers’ compensation insurance: Notable classes of employees excluded from mandatory work injury compensation insurance include employees earning more than $2600 per month in nonmanual work, government employees, and employees in certain industries, eg, retail, or certain companies, eg, banks
	Work Injury Compensation (Workers’ Fund) Regulations	Ex gratia payments: Provisions for the reimbursement of medical expenses incurred by employees for specific long-latency occupational diseases, eg, asbestosis, silicosis, mesothelioma. This applies specifically to cases where a work injury compensation claim was unsuccessful solely due to the employee contracting the disease after the statutory limitation period
**Japan**	Labour Standards Act	Liability for compensation: Employers must compensate employees for work injuries or illnesses at work. If equivalent payments are made under the Industrial Accident Compensation Insurance Act, the employer is exempt from the liability for providing compensation under this Act
	Industrial Accident Compensation Insurance Act	Provision of workers’ compensation insurance: The industrial accident compensation insurance is administered by the government Persons requiring workers’ compensation insurance: The insurance covers all businesses that hire workers, with exceptions such as businesses managed directly by the state, public agencies, or mariners Claims and compensation procedures: Methodology for the computation of compensation benefits stated
	Act on the Collection, etc. of Insurance Premiums of Labor Insurance	Provision of workers’ compensation insurance: The industrial accident insurance relationship is established on the date the business is commenced. Employers are required to notify the government within 10 days of establishment of the labor insurance relationship

### 3.2 Coverage and eligibility for workers’ compensation

#### 3.2.1 Singapore

##### 3.2.1.1 Work injuries

Singapore’s workers’ compensation system covers work injuries and occupational diseases that arise out of and in the course of employment. Notably, employers are not liable to compensate for work injuries sustained under the influence of alcohol, misuse of drugs, or deliberate self-injury or aggravation of injury by the worker.^12^

Transportation accidents to or from the workplace are covered under workers’ compensation only if the transport is operated by the employer or by a provider engaged by the employer, such as a chartered private bus. Transportation accidents arising from public transport services are not covered under the WICA.

##### 3.2.1.2 Occupational diseases

There are 38 occupational diseases covered by the workers’ compensation system that are specified in the Second Schedule of the WICA (see Appendix S1). They are grouped by exposure agent, target organ system, and occupational cancers. A general clause also classifies other diseases directly attributable to workplace exposure to chemical or biological agents as occupational diseases. There is a limitation period for compensation of occupational diseases, ranging from 1 to 3 years after the cessation of employment. For occupational diseases with a long latency period detected after the limitation period, such as silicosis, the affected worker may apply for financial assistance in the form of *ex gratia* payments under the Work Injury Compensation (Workers’ Fund) Regulations.^17^^,^^21^^,^^22^

While work-related mental health disorders and cerebrovascular diseases are not explicitly classified as occupational diseases under the WICA’s Second Schedule, workers have been compensated for conditions such as post-traumatic stress disorders, heart attacks, and strokes under the WICA by considering and assessing these conditions as injuries.^13^^,^^23^^,^^24^ However, it is unclear whether diseases of a more gradual onset, such as depression, are eligible for workers’ compensation.

##### 3.2.1.3 Types of workers covered

Under the WICA, workers’ compensation in Singapore covers any local or foreign employee under a contract of service or contract of apprenticeship.^25^ Additionally, with the introduction of the Platform Workers Act, platform workers are entitled to the same scope and level of work injury compensation under the WICA.^26^^,^^27^ However, exceptions include self-employed individuals, independent contractors, domestic workers, and uniformed personnel.

##### 3.2.1.4 Insurance coverage and financing framework

In Singapore, work injury compensation insurance is administered by MOM’s designated licensed private insurers. These insurers are required to comply with MOM’s compulsory terms.^28^ It is a requirement under the WICA for employers to provide workers’ compensation insurance for (1) all employees performing manual work, regardless of salary level, and (2) all employees performing nonmanual work, earning a salary of S$2600 or less a month.^29^ Under the WICA, it is also a requirement for platform operators to provide workers’ compensation insurance for all platform workers.^30^ For all other employees, workers’ compensation insurance is not a requirement and remains the prerogative of the employer.

Employers in Singapore are responsible for financing workers’ compensation insurance through designated insurers for workers who are required to be covered. Since 2021, the MOM has provided designated insurers with employers’ past workers’ compensation insurance claims data and workplace safety records for the purposes of implementing differentiated insurer premiums.^31^

#### 3.2.2 Japan

##### 3.2.2.1 Work injuries

In Japan, all injuries and illnesses sustained in the course of employment are covered under the LSA and IACIA. However, payment of IACIA proceeds for employment injuries may be restricted when (1) a worker intentionally causes an accident or (2) causes or worsens an injury or illness due to an intentional criminal act or serious error, or by not following instructions for medical care without valid reasons.^19^

Regarding commuting incidents, although workers are protected under the IACIA and workers’ compensation insurance, such incidents are not regarded as occupational accidents and do not entail employer responsibility under the LSA in relation to compensation liability or related dismissal protections. Commuting accidents covered include (1) travel between the worker’s residence and workplace, (2) travel between a workplace and another workplace, and (3) travel between a temporary residence and a family home for employees working away from family.^32^ Deviations from the travel route will not be treated as within the scope of commuting for work unless the disruption is minimal and necessary for daily life.

##### 3.2.2.2 Occupational diseases

Under Article 35 of the Enforcement Ordinance of the LSA, occupational diseases are classified into 11 categories based on exposure agent, target organ system, and occupational cancers, along with a general clause for all other diseases established to be work-related (see Appendix S2).^33^ While there is a limitation period for submission of claims, which ranges from 2 to 5 years, there is no restriction on claims eligibility after cessation of employment.

Circulatory diseases from overwork (*Karoshi*) are well-recognized in Japan, and a range of diseases are listed, including hemorrhagic and ischemic stroke, myocardial infarction, and other cardiovascular events such as angina.^34^ Mental health disorders were recognized as occupational diseases under the Ordinance for Enforcement of the LSA in 2010. “The Certification Criteria for Mental Illness due to Psychological Stress” were updated in 2023 to include “serious nuisance behavior by customers, business partners or facility users” as “customer harassment” under the Psychological Burden Evaluation Table for work-related factors, and recognized work-related causation for the aggravated component of a mental disorder when the worsening was attributable to a strong work-related psychological burden. A range of mental health conditions classified under F2 to F4 of the 10th International Classification of Diseases (ICD-10) are covered, including depression, anxiety, and post-traumatic stress disorder.^35^

##### 3.2.2.3 Types of workers covered

Under the LSA, “workers” refers to persons employed by a business and to whom wages are paid, regardless of the type of occupation. All workers employed by businesses are eligible to be compensated for industrial accidents and illnesses arising from work, with exceptions including mariners and domestic workers.^20^ While the LSA does not cover self-employed individuals, the IACIA has provisions to provide insurance coverage for these groups.^19^

##### 3.2.2.4 Insurance coverage and financing framework

The Japanese government administers workers’ compensation insurance and employers are required to make full payment for the premiums under the IACIA. The premium rates are calculated by multiplying the total amount of wages payable to workers in each workplace and the industry-specific premium rate. Of note, this industry-specific premium rate is dependent on the accident rate determined for the entire industry in general. Larger companies can participate in a merit system where premium rates are adjusted by up to 40% based on the amount of insurance benefits paid for work-related accidents and illnesses during the last 3 years.^19^^,^^36^^,^^37^

The IACIA is mandatory for all businesses that hire workers with exceptions, including persons insured by mariners’ insurance under the Mariners’ Insurance Act and certain government employees.^19^^,^^36^ There is a special voluntary enrollment scheme available for specific groups of workers including (1) business operators and family members helping out in small and medium-sized businesses who are not covered by the IACIA, (2) self-employed persons who engage in business without employing any workers, and (3) certain workers dispatched to foreign countries to work.^38^ Freelancers, including platform workers, are also eligible for special enrollment under the IACIA.^10^

### 3.3 Workers’ compensation benefits

#### 3.3.1 Singapore

Singapore’s workers’ compensation system provides 3 main benefits: (1) medical leave wages, (2) medical expenses, and (3) lump-sum compensation for permanent incapacity or death as summarized in Table 2. Workers who sustain an injury or occupational disease may claim compensation for temporary incapacity, which refers to being temporarily unable to perform work with reduced earnings, with compensation for medical leave wages and medical expenses. Permanent incapacity refers to residual incapacity that has stabilized and is unlikely to improve further, or an injury that results in permanent incapacity, for example, amputation of a limb. In general, claims for workers’ compensation have to be made within 1 year from the date of accident or diagnosis. This requirement extends to workers who wish to file for permanent incapacity.^40^^,^^41^

**Table 2:** Summary of benefits provided under Singapore’s WICA.

S/N	Compensation benefits	Brief description	Limitation period
**1**	Medical leave wages	This benefit provides for medical leave wages for working days covered by medical leave or light duty due to a work injury or disease, up to 1 year from the date of accident	1 year from the date of accident or diagnosis
**2**	Medical expenses	This benefit provides for expenses related to work accident for medical treatment received within 1 year from the date of the accident, or up to a maximum of $53 000, whichever is reached first This benefit can be used for rehabilitation costs and participation in return-to-work programs up to the specified limits^39^	1 year from the date of accident or diagnosis
**3**	Permanent incapacity or death	Permanent incapacity is based on a doctor’s assessment after the medical condition has stabilized and depends on the severity of the disability assessed	1 year from the date of accident or diagnosis

Abbreviations: WICA, Work Injury Compensation Act.

#### 3.3.2 Japan

The workers’ compensation system in Japan provides benefits across 9 categories, including benefits for (1) medical compensation, (2) temporary absence from work, (3) injury and disease pension, (4) disability, (5) surviving family benefits and funeral expenses, and (6) nursing care. Table 3 provides further information on each benefit.

**Table 3:** Summary of benefits provided under Japan’s IACIA

S/N	Compensation benefits	Brief description	Limitation period
**1**	Medical benefits	This benefit provides for medical treatment until the relevant work injury or illness is cured^a^	2 years from the date on which the medical expenses are made
**2**	Temporary absence from work benefits	This benefit starts from the fourth day on which a worker is unable to work and earn wages to receive medical treatment. The amount of compensation per day is equivalent to 60% of the basic daily payment amount	2 years after the worker becomes unable to work and earn wages for medical treatment
**3**	Injury and disease pension	This benefit provides for workers when a work-related injury or illness is not cured despite treatment for 1 year and 6 months and falls within the grades of illness or injury as specified by the MHLW	No claims procedures required. Determined by the chief of the relevant LSIO
**4**	Disability benefits	This benefit provides for work injuries and illnesses that have been cured with residual disability. Benefits provided depend on the severity of the disability (disability class), which is determined by the LSIO. Compensation payment for (3) will be subtracted from benefits paid	5 years from the day after the injury or illness is cured
**5**	Surviving family benefits and funeral expenses benefits	This surviving family benefits (lump sum or pension) is provided for the surviving family of a worker who dies as a result of work The funeral expenses benefits reimburse funeral expenses for the individual responsible (family or employer) for the funeral of a worker who dies as a result of work	5 years from the day after the worker’s death for surviving family benefits 2 years from the day after the worker’s death for funeral expenses
**6**	Nursing care benefits	Nursing care compensation benefits are paid to workers who require nursing care due to a disability. Workers are compensated for constant nursing or on-call nursing care depending on severity of the disability	2 years from the first day of the month following the month when nursing care was received

Abbreviations: IACIA, Industrial Accident Compensation Insurance Act; LSIO, Labour Standards Inspection Office; MHLW, Ministry of Health, Labour and Welfare.

^a^Under the IACIA, “cured” refers to a state where the symptoms are stabilized and where no further medical effect can be expected despite further recognized medical treatment.

While there is a statute of limitation of 2 to 5 years for insurance claims, there is no limitation period from when the worker left employment, allowing workers with long-latency diseases to claim compensation under the IACIA. There is also no specified limit on the duration of medical expense coverage for work injuries and illnesses.^19^^,^^42^ However, under Article 81 of the LSA, if a worker who sustained a work-related injury or illness fails to recover within 3 years of commencing medical treatment, the employer may choose to pay compensation equivalent to 1200 days of the worker’s average wage in lieu of continued compensation.^20^

### 3.4 Claims process, dispute resolution, and compensation statistics

#### 3.4.1 Singapore

##### 3.4.1.1 Process for filing for workers’ compensation

The process for workers’ compensation in Singapore begins with the worker seeking medical treatment with any medical practitioner and informing the employer of the work injury sustained or occupational disease diagnosed. The MOM is notified of the incident by the employer or worker, which triggers the claims process. For workers covered by workers’ compensation insurance, the case will be processed and assessed by the respective designated insurers in accordance with the MOM’s guidelines.^28^ For workers not covered by workers’ compensation insurance, the claim will be processed and assessed by the MOM and employers will have to pay compensation if a valid claim is made.^39^ Where necessary, designated insurers may refer to medical expertise and specialized opinions to assess the claims.

Upfront payment is required for medical expenses incurred for work injuries and occupational diseases. Employers are required to pay for workers’ medical leave wages and medical expenses first, while awaiting reimbursement from designated insurers. For nonemergency cases involving migrant workers, hospitals may request a letter of guarantee from the employer before treatment to ensure that the employer is aware and able to pay for the medical expenses.^43^ In Singapore, the duration for claims processing takes a median time of about 6 months by designated insurers.^44^

##### 3.4.1.2 Dispute management

Workers, employers, and designated insurers can dispute workers’ compensation claims relating to compensation amount, permanent disability assessment, or work-relatedness of the injury by filing an objection notice with MOM. For disputes regarding permanent disability assessment, the injury will be reassessed by a medical board convened by the MOM comprising specialists from restructured hospitals, with the board’s assessment taken as final. Other disputes are addressed through a pre-hearing conference held by the MOM. If resolution is not achieved at the conference, the case is referred to the Labour Court for a hearing.^45^^,^^46^

Measures are in place to protect workers from retaliation by employers. Workers may file a claim with the MOM to assist with disputes regarding compensation claims.^46^ For migrant workers, employers are not allowed to send these workers home against their wishes if there is an outstanding claim under the WICA.^47^

##### 3.4.1.3 Compensation data

**Figure 2:**
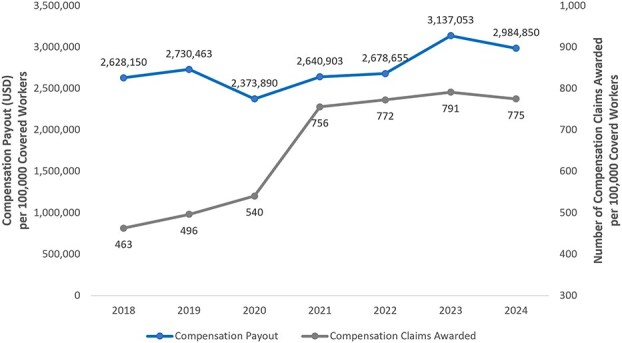
Trends in compensation claims awarded and compensation payout in Singapore from 2018 to 2024.

The number of workers’ compensation claims awarded per 100 000 covered workers in Singapore has been on an increasing trend, from 463 cases in 2018 to 775 cases in 2024, as seen in Figure 2. A sharp increase from 540 cases in 2020 to 756 cases in 2021 was due to changes in reporting requirements, which required employers to report work-related incidents resulting in any medical leave or light duties. In terms of compensation payout per 100 000 covered workers, this has shown a general increasing trend from USD 2.63 million in 2018 to USD 2.98 million in 2024.^48^

#### 3.4.2 Japan

##### 3.4.2.1 Process for filing for workers’ compensation

Japan’s workers’ compensation system is a public insurance scheme operated by the national government and claims are not reviewed by private insurers. The claims process begins with treatment at a medical facility, which can be designated by the IACIA, known as Rosai Hospitals, or at other medical facilities.^42^ Workers treated at a Rosai Hospital can receive treatment under their compensation benefits without upfront payment, whereas workers treated at other medical facilities are required to pay upfront before subsequently claiming under the IACIA.^49^

Claims for compensation under Japan’s system are, in principle, initiated by the injured worker or the bereaved family. When a claim is filed with the Labour Standards Inspection Office (LSIO), the LSIO investigates and makes an administrative determination as to whether the claim is work-related. Where necessary, the LSIO may refer to medical expertise and specialized opinions before making its decision in accordance with official recognition criteria issued by the MHLW. Once certified as occupational in nature, the LSIO authorizes payment of insurance benefits.^50^ In Japan, it generally takes 1 month for processing of claims for medical expenses and temporary absence from work, while claims for disability benefits generally take 3 months.^42^

##### 3.4.2.2 Dispute management

Where a worker or an employer disagrees with either the determination of work-relatedness, the approval or rejection of benefit claims, or the assessment of the degree of permanent disability, the Japanese system has a multitiered system of administrative and judicial review. A party may file (1) a request for administrative review against the decision of the LSIO; (2) a request for re-examination against the decision of the Labour Insurance Review Officer at the Prefectural Labour Bureau; and (3) a further appeal to the Labour Insurance Appeal Committee.

If the dispute remains unresolved, it is possible to appeal the case via a civil suit.^37^^,^^51^ Judicial review is conducted under Japan’s 3-tier court system, ensuring judicial oversight of administrative determinations under the workers’ compensation scheme. To protect workers from retaliation, the LSA prohibits dismissal of a worker who is absent from work for medical treatment due to an occupational injury or illness or within 30 days thereafter.^20^

##### 3.4.2.3 Compensation data

**Figure 3:**
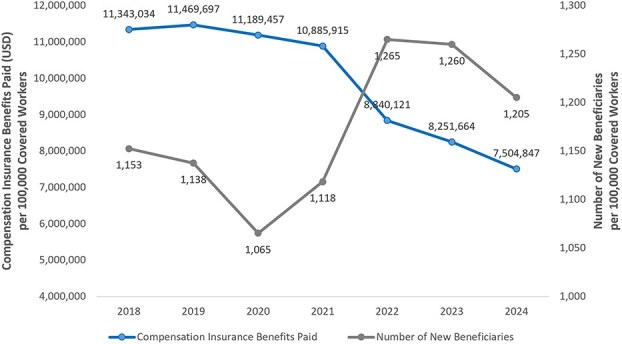
Trends in number of new beneficiaries and compensation insurance benefits paid in Japan from 2018 to 2024.

In Japan, the number of new beneficiaries per 100 000 covered workers showed a decrease, from 1153 in 2018 to 1065 in 2020, before increasing to 1265 in 2022, as seen in Figure 3. The increase in new beneficiaries from 2020 to 2022 may partly be attributable to the recognition of COVID-19 cases as work-related accidents. Compensation insurance benefits, paid in Japanese yen (JPY) per 100 000 covered workers, decreased slightly from JPY 1.25 billion in 2018 to JPY 1.14 billion in 2024. The decline was more marked in USD terms, from USD 11.34 million to USD 7.50 million, partly due to the weakening of the JPY against the USD.

### 3.5 Role of the occupational health physician for workers’ compensation

Occupational health physicians may provide occupational health services to companies as direct employees or through consulting agreements. In Singapore, occupational health services are provided predominantly through consulting agreements, where physicians support companies externally rather than as direct employees. In Japan, while part-time consulting agreements are more common, occupational health physicians may also be directly employed as company employees, especially in larger companies.

Additionally, occupational health physicians may provide independent medical opinions to parties responsible for the assessment of workers’ compensation claims and participate in government-level medical committees to determine work-relatedness for complex cases, as seen in Japan. A small proportion of occupational health physicians in both Japan and Singapore also choose to work for regulators at the national level, to provide medical and occupational health expertise in the development of policies and regulations.

#### 3.5.1 Singapore

All physicians registered with a license to practice in Singapore are responsible for diagnosing occupational diseases and workplace injuries. This is not exclusively limited to occupational health physicians. Additionally, notification of occupational diseases to the MOM and assessment of the percentage permanent incapacity for lump sum compensation must be carried out by diagnosing physicians,^12^^,^^52^ including occupational health physicians, who may act either as an independent assessor or as a clinical service provider under contractual agreements with the employer.

Upon the diagnosis and notification of the occupational disease, the diagnosing physician has a duty to communicate the diagnosis to employers, for employers to fulfill their statutory reporting duties to MOM. While designated insurers make the final decisions on admissibility and compensation amounts on behalf of MOM, these decisions depend heavily, if not entirely, on the diagnosing physician’s clinical assessment of the diagnosis and the extent of permanent disability.

#### 3.5.2 Japan

In Japan, the institutional separation between occupational health practice and workers’ compensation adjudication is reinforced by legal and administrative frameworks governing the independence of occupational physicians. Under Article 14 of the Ordinance on Industrial Safety and Health, occupational health physicians are responsible for investigating the causes of workers’ health disorders and proposing measures to prevent their recurrence. They have no statutory authority within the workers’ compensation system to determine work-relatedness of a condition, and are not required to notify or submit opinions to the MHLW for workers’ compensation purposes.

While occupational health physicians may assess the work-relatedness of health conditions or provide medical opinions on occupational diseases, such assessments are conducted to advise employers on appropriate work accommodation and preventive measures. They do not impact admissibility of claims, compensation amounts, disability certification, or determinations of work-relatedness within the social security system. The LSIO makes and adjudicates the final decision on admissibility of claims and compensation amounts.

## 4. Discussion

### 4.1 What are the key factors shaping the design of workers’ compensation systems?

In comparing Singapore and Japan, there are several key factors that play an important role in shaping workers’ compensation systems.

First, Singapore and Japan differ in their social welfare framework and policies, which may influence the extent of coverage for occupational diseases and commuting accidents. Singapore’s model of social welfare is guided by 3 principles—self-reliance, family as the first line of support, and a many-helping-hands approach.^53^ These principles emphasize the use of personal, family, and community resources before relying on the government for assistance. The model has also been described as “productivist,” serving to support economic development.^54^^,^^55^ Taken together, this explains the tightly scoped approach to workers’ compensation to provide workers with basic financial protection and coverage while ensuring economic competitiveness and limiting costs to businesses. In contrast, Japan’s social welfare system is anchored in its constitution to “maintain the minimum standards of wholesome and cultured living” and to “promote and expand social welfare, social security and public health services to cover every aspect of the life of the people.”^56^ Correspondingly, Japan’s workers’ compensation system is in line with its approach to social welfare by prioritizing worker health with comprehensive compensation benefits and coverage.

Second, demographic factors of the working population could also explain differences in the extent of coverage provided by workers’ compensation systems. Singapore relies significantly on foreign workers in industries such as construction, shipyard, and manufacturing,^55–59^ which is in stark contrast to Japan’s labor market, which comprises a predominantly resident workforce, with foreigners accounting for only 3% of Japan’s employed population in 2024.^60^ In Singapore, semi-skilled roles are largely filled by migrant workers, and employers may view this workforce as one with a limited tenure that is easier to replace.^61^ This may in turn weaken incentives to implement more comprehensive workers’ compensation benefits. On the other hand, Japan’s predominantly local workforce creates stronger incentives to provide comprehensive benefits that safeguard worker health and support preventive measures to reduce potential spillover costs to health care and social support systems.

Finally, structural differences in Singapore and Japan’s systems have influenced how workers’ health and labor issues are approached. In Singapore, health and labor issues are overseen by separate ministries, namely the Ministry of Health (MOH) and the MOM, and private designated insurers insure and process claims. This disperses functions across institutions and corresponds to a fragmented workers’ compensation system with greater barriers to seeking treatment and compensation. In contrast, Japan’s MHLW oversees both health and labor issues, is responsible for workers’ compensation insurance, and the MHLW’s LSIO processes compensation claims. Although the Japanese workers’ compensation system reflects an integrated approach to health and labor, integration does not eliminate the need for resource allocation and budgetary prioritization of workers’ compensation system against competing priorities, such as an aging workforce and unemployment support.

### 4.2 To what extent should occupational health physicians be involved in workers’ compensation?

In both Singapore and Japan, occupational health physicians are not directly responsible for the clinical adjudication of compensation claims. Occupational health physicians possess expertise in causation assessment and understand the complex relationship between workplace exposures and health outcomes. Although this expertise is shared, the institutional function assigned to clinical assessments by occupational health physicians differs. Singapore’s workers’ compensation system generally places more weight on the occupational health physician’s assessment towards the final determination of work-relatedness, disability, and compensation payout compared with Japan. To what extent, then, is it appropriate for occupational health physicians to assume a more substantive role in determining and affecting the admissibility of workers’ compensation claims and quantum of compensation?

A principal consideration is the employment structure of occupational health physicians. In Japan, larger companies hire occupational health physicians as full-time company employees with an ongoing employment relationship that may create an inherent structural conflict of interest. While the MHLW has safeguards in place to protect the independence of occupational health physicians and requires employers to report their resignation or dismissal to the health and safety committee,^62^ occupational health physicians remain dependent on the employer for economic security. A perception of bias, regardless of actual conduct, could undermine worker trust and such structural considerations may explain the emphasis on maintaining institutional separation between occupational health practice and workers’ compensation adjudication in Japan.

In Singapore, companies typically contract occupational health service providers, creating an indirect financial relationship. While occupational health physicians in Singapore have greater organizational distance from employers, potential conflicts of interest remain, and physicians may face pressure to maintain favorable relationships with employers to secure contract renewals. Nevertheless, this arm’s-length relationship generally affords greater independence, as physicians serve multiple clients rather than depending on a single employer. This greater structural independence may explain why more weight is given to the occupational health physician’s assessment for the determination of admissibility of claims and compensation amounts in Singapore.

In Japan’s system, where some occupational health physicians are directly employed by larger companies, independent medical panels or mandatory second opinions from external occupational health physicians may be considered for disability assessments above certain thresholds or for contested claims. In Singapore’s model, clear contractual provisions should protect physician independence from adverse consequences for assessments favorable to workers. While structural differences between countries necessitate jurisdiction-specific approaches, it may be prudent to maintain the current division of responsibility, where occupational health physicians provide medical assessments and recommendations, while the authorities determine final compensation.

### 4.3 Is there distributive fairness in the worker–employer–society dynamics?

Workers’ compensation systems aim to provide injured workers with financial assistance and support dependents in the event of death.^9^ The degree and extent to which these aims are legislated and regulated depends on the interplay among 3 main parties: (1) the worker, (2) the employer, and (3) society at large. An ideal workers’ compensation system seeks to distribute and balance the costs and benefits equitably, and we examine how the respective systems in Singapore and Japan are configured to achieve distributive fairness.

#### 4.3.1 Worker

In general, the compensation systems of Singapore and Japan cover an adequate range of workers and provide baseline protection for workers against retaliation by employers. However, Japan’s system provides a wider coverage of occupational diseases with less restrictive limitation periods, more comprehensive compensation benefits, and coverage of all commuting work accidents, with processes in place to reduce barriers for workers seeking treatment and compensation. Fundamentally, this reflects a different legislative philosophy and divergence between the 2 countries on the boundary of where employment risk lies. From a workers’ fairness standpoint, Singapore’s system appears to disadvantage workers who have to rely on supplementary private insurance or broader social protection mechanisms. The extent to which Singapore’s existing social protection mechanisms sufficiently address these residual vulnerabilities, especially for lower-wage migrant workers, remains an open question for further policy discussion.

In Singapore, the delegation of workers’ compensation claims processing and assessment to private designated insurers by the MOM has important implications for workers. These insurers take on dual roles: they sell workers’ compensation insurance policies and generate premium revenue, while simultaneously adjudicating workers’ compensation claims. This raises a potential conflict of interest as insurers are effectively assessing and approving their own liabilities, with a potential economic incentive to minimize the number of claims approved or compensation amounts awarded. While there are safeguards by the MOM, including issuance of guidelines to insurers on claims processing standards and provision of dispute resolution pathways for workers and employers, these measures may be viewed as inadequate, with limitations in addressing the fundamental conflict of interest. Guidelines and monitoring of insurers’ compliance remain opaque to the public, with limited available data to enable assessment of systemic biases, including details of denied claims, average processing times, and outcome variations between different insurers. Furthermore, dispute resolution pathways are primarily downstream in nature, and navigating the compensation system can be challenging for workers.

Perhaps Singapore could consider alternative structural arrangements in the processing of workers’ compensation claims. Routine independent medical review before claim decisions are finalized, either by independent occupational health physicians or governmental officials as in Japan’s system, may help to address the conflict of interest and strengthen upstream safeguards. For claims involving permanent disability where compensation amounts are deemed more significant, insurers should be required to obtain medical assessments from independent occupational health physicians or specialists without an ongoing commercial relationship. These occupational health physicians or specialists may be selected and managed by the regulator. Systematic auditing of insurer decisions and publication of insurer-specific performance metrics could support monitoring and detection of inappropriate claim handling. While these recommendations can facilitate fair and equitable outcomes that prioritize fairness for workers, they involve trade-offs such as administrative costs and governmental capacity, which should be evaluated carefully.

#### 4.3.2 Employers

Both Singapore and Japan have systems in place to ensure fair processing of workers’ compensation claims. However, Singapore’s system generally places a lower administrative burden and cost on employers. Employers are required to obtain workers’ compensation insurance only for a subgroup of workers considered more vulnerable,^63^ while limitation periods for occupational diseases after cessation of employment reduce companies’ potential long-term liability. In general, Singapore’s system is designed more narrowly around employer-controlled risks, where the employer bears liability primarily for circumstances over which they have meaningful control, as evident in the more restrictive commuting accident coverage. This avoids placing an inequitable burden on employers, especially for smaller employers, with broader societal policies addressing the residual risks.

In Japan, insurance coverage is mandatory for all businesses that hire workers, with no restriction on claims eligibility after cessation of employment. Correspondingly, employers are faced with higher costs of insurance premiums and administrative burden in the renewal and payment of workers’ compensation insurance. This is a policy choice that is consistent with Japan’s approach to social welfare in prioritizing the health and well-being of the workers.

#### 4.3.3 Society

Employers may not always address the negative externalities of business activities including the costs of ill health and disability, which fall upon individuals, families, and society at large.^64^ Workers’ compensation systems pool the risk of work-related ill health and incentivize employers to improve standards of WSH.

Both Japan and Singapore utilize differentiated workers’ compensation insurance premiums to signal to companies the true costs of poor WSH. Japan’s system is transparent and encourages prioritization of WSH through a merit-based system, whereby premium rates are adjusted according to the amount of insurance benefits paid for work-related incidents.^36^ In Singapore, although the MOM provides designated insurers with employers’ past claims and safety records,^31^ it remains unclear how designated insurers apply this information to differentiate premiums. Consequently, employers may find it difficult to evaluate potential cost savings and benefits of prioritizing occupational health and safety.

In summary, the contrasting models of Singapore and Japan offer valuable insights on distributive fairness. Singapore’s compensation system focuses on ensuring employer competitiveness while providing workers with basic financial protection and limiting spillover costs to society through tightly scoped compensation coverage and benefits. Japan’s system prioritizes worker health with more comprehensive compensation benefits and coverage, and a clear mechanism to recognize the true costs of poor health and safety at the workplace. Correspondingly, higher costs and administrative burden are placed on businesses in Japan.

### 4.4 What are the common challenges for workers’ compensation systems in Singapore and Japan?

#### 4.4.1 Apportioning attributability of occupational diseases for workers’ compensation

A common challenge for Singapore and Japan is determining the attributable proportion to work for occupational diseases that are multifactorial in nature. For example, work-related heart attacks may be contributed to by age, genetics, and lifestyle factors apart from work-related contributions. In both systems, admission of claims for occupational diseases follows a binary approach—once a claim is accepted, compensation amounts are not apportioned based on the degree of work-related contribution. Further research and policy approaches to determining the apportionment of work-relatedness in occupational disease compensation claims may help enhance fairness for both employers and employees, ensuring a more balanced and equitable distribution of compensation and responsibility.

#### 4.4.2 Underreporting of work-related incidents

In both Singapore and Japan, underreporting of work-related incidents remains a significant concern.^65^^,^^66^ Workers may lack awareness regarding reporting procedures, fear repercussions on their employment, or be deterred by the perceived complexity and effort involved in filing a claim. Employers may likewise underreport incidents to maintain a favorable WSH record.

In Singapore, underreporting may be more prevalent among workers not covered by workers’ compensation insurance as employers are directly responsible for the costs of compensation. A unique challenge observed in Japan is the tendency for workers to claim personal health insurance benefits for work-related incidents instead of seeking workers’ compensation.^36^ This misclassification may contribute to an underestimation of occupational risks, hinder efforts to improve WSH, and unfairly place the burden of work-related incidents on society. The extent of underreporting in Singapore and Japan remains unclear, and there may be value in conducting studies to understand the scale of underreporting, identify affected worker groups, and develop targeted interventions with monitoring and evaluation to assess their effectiveness.

## 5. Conclusions

In conclusion, both Singapore and Japan have robust workers’ compensation systems built upon strong legal frameworks, with a shared commitment to safeguarding workers’ health. While there are differences in the approach taken for workers’ compensation in both countries, there is potential for collaboration and learning. Understanding the underlying principles and trade-offs in each system allows us to identify opportunities to strengthen fairness and ensure more equitable outcomes. By sharing best practices, Singapore and Japan can better address challenges such as underreporting, and work on improving understanding of the attribution of work to occupational diseases.

## Supplementary Material

Supplementary_materials_uiag038

## Data Availability

Data for the study were extracted from published scientific literature and publicly available data sources. Derived data are available from the corresponding author upon reasonable request.
