# Supplementary material for: Comparison of workers’ compensation systems in Singapore and Japan
Source: J Occup Health. 2026 Jul 12;68(1):uiag038. doi: 10.1093/joccuh/uiag038 (PMC13464671; doi:10.1093/joccuh/uiag038)
Supplement: Supplementary file 1 [file supplementary_materials_uiag038.zip › Annex A Singapore, Second Schedule of Work Injury Compensation Act 2019.docx]

**Annex A – Singapore, Second Schedule of Work Injury Compensation Act 2019**

**OCCUPATIONAL DISEASES**

|  | **Diseases caused by physical agents** |
| --- | --- |
| 1. | Barotrauma |
| 2. | Cataract due to infra-red, ultraviolet or ionising radiation |
| 3. | Compressed air illness or its sequelae, including dysbaric osteonecrosis |
| 4. | Diseases caused by excessive heat |
| 5. | Diseases caused by ionising radiation |
| 6. | Noise-induced hearing loss |
|  | **Diseases caused by chemical agents** |
| 7. | Poisoning by aniline |
| 8. | Poisoning by arsenic |
| 9. | Poisoning by benzene or a homologue of benzene |
| 10. | Poisoning by beryllium |
| 11. | Poisoning by cadmium |
| 12. | Poisoning by carbamates |
| 13. | Poisoning by carbon disulphide |
| 14. | Poisoning by carbon monoxide gas |
| 15. | Poisoning by cyanide |
| 16. | Poisoning by halogen derivatives of hydrocarbon compounds |
| 17. | Poisoning by hydrogen sulphide |
| 18. | Poisoning by lead |
| 19. | Poisoning by manganese |
| 20. | Poisoning by mercury |
| 21. | Poisoning by organophosphates |
| 22. | Poisoning by oxides of nitrogen |
| 23. | Poisoning by phosphorus |
| 24. | Toxic anaemia |
| 25. | Toxic hepatitis |
|  | **Diseases caused by biological agents** |
| 26. | Anthrax |
| 27. | Glanders |
| 28. | Leptospirosis or its sequelae |
| 29 | Occupational infectious disease |
|  | **Musculoskeletal disorders** |
| 30. | Work-related musculoskeletal disorder |
|  | **Respiratory disorders** |
| 31. | Asbestosis |
| 32. | Byssinosis |
| 33. | Occupational asthma |
| 34. | Silicosis |
|  | **Skin disorders** |
| 35. | Occupational skin disease |
|  | **Cancers (Malignant diseases)** |
| 36. | Malignant mesothelioma |
| 37. | Occupational liver angiosarcoma |
| 38. | Occupational skin cancer |
