# Supplementary material for: Comparison of workers’ compensation systems in Singapore and Japan
Source: J Occup Health. 2026 Jul 12;68(1):uiag038. doi: 10.1093/joccuh/uiag038 (PMC13464671; doi:10.1093/joccuh/uiag038)
Supplement: Supplementary file 1 [file supplementary_materials_uiag038.zip › Annex B Japan, Table 1-2 of the Ordinance for Enforcement of the Labor Standards Act (Ordinance of the Ministry of Health and Welfare No. 23 of 1947).docx]

**Annex B – Japan, Table 1-2 of the Ordinance for Enforcement of the Labor Standards Act (Ordinance of the Ministry of Health and Welfare No. 23 of 1947)**

| (i) | Disease resulting from injuries incurred in the course of employment |
| --- | --- |
| (ii) | The following disease due to physical factors:   a) Disease in the anterior part of the eye or skin disease due to work involving exposure to ultraviolet rays b) Eye disease such as retinal burn and cataract or skin disease due to work involving exposure to infrared rays c) Eye disease such as retinal burn or skin disease due to work involving exposure to laser beams d) Eye disease such as cataract due to work involving exposure to microwaves e) The following disease due to work involving exposure to ionizing radiation: radio dermatitis such as acute radiation disease and skin ulcer, eye disease due to radiation such as cataract, radiation fibrosis of the lung, hematopoietic disorder such as aplastic anemia, osteonecrosis, and other illness due to radiation f) Caisson disease or diver's disease due to work in high-pressure rooms or diving g) Mountain sickness or aircraft dysbarism due to work done in low- pressure places h) Heat stroke due to work done in hot places i) Burns due to work to handle extremely heated materials j) Frostbite due to work done in cold places or to handle cold materials k) Hearing disability such as deafness due to work done in noisy places l) Necrosis such as of finger tissues due to work involving exposure to supersonic waves m) In addition to the disease prescribed in (a) to (l) inclusive their annexed disease and other disease which clearly result from work involving exposure to physical factors |
| (iii) | The following disease caused by a form of work which involve extreme physical tension:   a) Muscle, tendon, bone, or joint disease or prolapse of internal organs due to strenuous work b) Low back pain due to work to handle heavy objects, those done in unnatural postures or others which involve excessive tension to low back c) Peripheral circulatory disorder, peripheral nerve disorder, or motive organ disorder of fingers or forearm etc. due to work which vibrate the body due to use of equipment or machinery such as rock drill riveter, or chain saw d) Motive organ disorder of the back of the head, neck, shoulder girdle, upper arm, forearm, or fingers due to work which require repeated input into a computer or other operation involving excessive tension on the upper limbs e) In addition to the illness listed in (a) to (d) inclusive their annexed disease and other that are clearly caused by work executed in ways which involve excessive tension to the body |
| (iv) | The following disease due to chemical substances etc:   a) Disease designated by the Minister of Health, Labour and Welfare due to work involving exposure to simple chemical substances or compounds (including alloys) designated by the Minister of Health, Labour and Welfare b) Inflammation of mucous membranes of the eyes or respiratory troubles such as inflammation of mucous membranes of the respiratory organs due to work involving exposure to pyrolytic products of synthetic resins such as of fluoric resin, vinyl chloride resin, or acrylic resin c) Skin disease due to work involving exposure to soot, mineral oil, lacquer, tar, cement, or amine type resin hardeners manufactured or some other equivalent d) Skin disease conjunctivitis, or respiratory disease such as rhinitis or asthma bronchiale due to work involving exposure to proteolytic enzymes e) Respiratory disease such as allergic rhinitis or asthma bronchiale due to work done in places where particulate of lumber or fur or some other equivalent are scattered or those involving exposure to antibiotics or some other equivalent f) Respiratory disease due to work done in places where particulate of cotton waste or some other equivalent are scattered g) Benign asbestos pleural effusion or diffuse pleural thickening due to work involving exposure to asbestos h) Anoxia due to work done in places with low oxygen content i) In addition to the disease listed in (a) to (h) inclusive their annexed disease and other disease that are clearly caused by work involving exposure to chemical substances |
| (v) | Pneumoconiosis or disease listed in the items of Article 1 of the Ordinance for Enforcement of the Pneumoconiosis Act (Ordinance No. 6 of the Ministry of, Labor 1960) which are complication of pneumoconiosis prescribed by the Pneumoconiosis Act (Act No. 30, 1960) due to work done in places where fine particulate is scattered. |
| (vi) | The following disease due to pathogens such as bacteria and viruses:   a) Infectious diseases due to treating, nursing or caring patients or work to handle pathogens for research and other purposes b) Infectious diseases such as brucellosis and anthrax due to work to handle animals and their carcasses, fur, hide, other animal materials, or rags and other old cloth c) Leptospirosis such as Weil disease due to work in damp places d) Tsutsugamushi disease due to outdoor work e) In addition to the disease listed in (a) to (d) inclusive their annexed disease and others that are clearly caused by work involving exposure to pathogens such as bacteria and viruses |
| (vii) | The following disease due to carcinogen, carcinogenic agent, or work done in the carcinogenic processes:   a) Tumor of urinary tract due to work involving exposure to benzidine b) Tumor of urinary tract due to work involving exposure to betanaphthylamine c) Tumor of urinary tract due to work involving exposure to 4-aminodiphenyl d) Tumor of urinary tract due to work involving exposure to 4-nitrodiphenyl e) Lung cancer due to work involving exposure to bis (chloromethyl) ether f) Lung cancer due to work involving exposure to benzotrichloride g) Lung cancer or mesothelioma due to work involving exposure to asbestos h) Leukemia due to work involving exposure to benzene i) Angiosarcoma of liver or hepatocellular carcinoma due to work involving exposure to vinyl chloride j) Leukemia, lung cancer, skin cancer, osteosarcoma, thyroid carcinoma, multiple myeloma, or non-Hodgkin lymphoma due to work involving exposure to ionizing radiation k) Tumors of urinary tract due to work the auramine manufacturing process l) Tumors of urinary tract due to work in the magenta manufacturing process m) Lung cancer due to work in the coke or producer gas manufacturing process n) Lung cancer or cancer in the upper respiratory organs due to work in the chromate or bichromate manufacturing process o) Lung cancer or cancer in the upper respiratory organs due to work in the nickel smelting or refining process p) Lung or skin cancer due to work in the processes to manufacture or refine metals using ores containing arsenic or those in inorganic arsenic compounds in manufacturing process q) Skin cancer due to work involving exposure to soot, mineral oil, tar, pitch, asphalt, or paraffin r) In addition to the disease listed in (a) to (q) inclusive their annexed disease and others that are clearly caused by work involving exposure to carcinogen, carcinogenic agent, or work done in the carcinogenic processes |
| (viii) | Cerebral hemorrhage, subarachnoid hemorrhage, cerebral infarction, hypertensive encephalopathy, myocardial infarction, angina pectoris, cardiac arrest (including sudden cardiac death), dissecting aneurysm of the aorta, or their annexed disease due to work which require extended hours of work over a long period of time or other work which significantly worsens a vascular lesion, etc. |
| (ix) | A mental and behavioral disorder or their annexed disease due to life-threatening accidents or jobs involving excessive mental tension |
| (x) | In addition to the disease listed in the preceding items those designated by the Minister of Health, Labour and Welfare. |
| (xi) | Other disease that is clearly caused by operations. |
